# Sex- and gender-related aspects of patient safety in radiation oncology: a scoping review

**DOI:** 10.3389/fonc.2026.1962214

**Published:** 2026-10-27

**Authors:** Maximilian Grohmann, Maike Trommer, Angela Besserer, Andrea Baehr

**Affiliations:** 1 Department of Radiooncology, University of Leipzig Medical Center, Leipzig, Germany; 2 Department of Radiotherapy and Radiation Oncology, University Medical Center Hamburg-Eppendorf, Hamburg, Germany; 3 Department of Radiation Oncology, University Hospital Bonn, Bonn, Germany; 4 Department of Radiation Oncology, Ernst von Bergmann Hospital Potsdam, Potsdam, Germany

**Keywords:** gender, patient safety, PRISMA-ScR, radiation oncology, radiotherapy, scoping review, sex

## Abstract

**Background:**

Radiation oncology is a safety-critical clinical discipline in which sex-related biological attributes and gender-related social processes may influence treatment planning, treatment effects, symptoms, care use, and follow-up. Their relevance to patient safety across the radiation-oncology pathway remains incompletely characterized. We examined evidence for sex- or gender-related aspects affecting safety in radiation oncology.

**Methods:**

We conducted a scoping review of original studies identified through a PubMed search conducted on 22 July 2026 and through backward reference-list searching. Data were charted at the level of individual findings, classified according to the operationalization of sex and gender, and mapped to four published patient-safety categories and 27 subcategories. Synthesis was descriptive; no meta-analysis, formal critical appraisal, or certainty assessment was undertaken.

**Results:**

The PubMed search yielded 159 records. Thirty-two database-identified studies and five citation-derived original studies met the eligibility criteria. The 37 included studies contributed 49 reportable empirical findings: 33 differences or associations, 10 mixed findings, and six statistically non-significant findings. Forty-four findings were clinical or technical outcome signals with potential safety relevance and five directly concerned care processes. Evidence mapped to six patient-specific subcategories. Twenty studies were classified as analyses of sex and 17 as unclear; no study operationalized multidimensional gender.

**Conclusions:**

The targeted search, including five citation-derived studies, may selectively capture studies reporting sex only as a covariate. The retrieved evidence indicates that sex can be relevant to the safe design and delivery of radiotherapy, but the evidence is fragmented, predominantly observational, and largely limited to clinical outcome associations. Prospective studies should test whether clearly defined sex- and gender-informed care processes improve safety.

## 1 Introduction

Radiation oncology (RO) is a safety-critical discipline in which complex technology is embedded in a longitudinal clinical pathway. Safe care depends not only on correct imaging, contouring, treatment planning, quality assurance, and dose delivery, but also on appropriate indication, communication, supportive care, medication management, recognition of adverse effects, treatment completion, and follow-up. The PaSaGeRO systematic review published a patient-safety framework that organizes the radiation-oncology pathway into four categories and 27 subcategories spanning human resources, institutional culture, quality and risk management, and patient-specific processes; the subsequent PaSaRO multi-method study further developed the related questionnaire and assessed its content validity (1, 2). This breadth is useful because clinically relevant harm may arise even when the technical treatment itself has been delivered as intended.

Following the World Health Organization, patient safety concerns preventing avoidable harm and minimizing unnecessary risks from health care. For this review, safety relevance meant a potential implication for preventing, detecting, or managing harm along the radiotherapy pathway; an outcome association alone did not establish preventability^^1^^.

Sex and gender may influence several components of this pathway, but they are not interchangeable concepts. Sex refers to biological attributes such as anatomy, hormones, chromosomes, and sex-related physiology, whereas gender encompasses identities, roles, relations, behavior, and structural conditions (3). In oncology, sex-related variation may affect body composition, organ exposure, pharmacology, tissue response, and susceptibility to acute or late effects (4, 5). Gender-related processes may influence presentation, communication, symptom reporting, treatment choices, medication use, rehabilitation, and access to supportive services (6–8). Female sex has also been associated with complementary-medicine use in a systematic review of cancer populations (9). These mechanisms may interact with age, tumor site, comorbidity, treatment intensity, and socioeconomic position, making a simple female-versus-male comparison difficult to interpret (4, 6, 8).

Publications frequently use sex and gender synonymously, even when the measured variable is a binary demographic field and no gender construct has been defined. The SAGER guidelines therefore recommend transparent reporting of how sex and gender were defined, measured, analyzed, and interpreted (3). Without this distinction, biological and social explanations can be conflated, and apparent differences may be attributed to sex or gender when they instead reflect diagnosis, treatment selection, body composition, or other confounding factors.

Previous reviews have described female–male differences in radiosensitivity, treatment tolerance, and radiotherapy effectiveness, while emphasizing heterogeneous methods, confounding, and limited separation of biological sex from gender-related influences (4, 10, 54). This literature has largely been organized by tumor entity, treatment efficacy, or toxicity rather than by the clinical processes through which hazards are anticipated, detected, and managed. It therefore remains unclear which reported differences constitute patient-safety signals, where along the radiotherapy pathway they arise, and whether any sex- or gender-informed care process improves safety. The present review differs from previous narrative syntheses by classifying how sex and gender were operationalized, and mapping findings at the level of care processes and safety-relevant outcomes.

Within this review the working groups on patient safety and women in RO of the German Society of RO (DEGRO) conducted a scoping review to identify and characterize the evidence on sex- or gender-related differences with potential relevance to patient safety in radiation oncology and to map the reported findings to the published 27-subcategory patient-safety framework.

## 2 Methods

### 2.1 Review design

We conducted a scoping review using established methodological guidance (11, 12). A scoping approach was selected to map the extent, characteristics, and distribution of heterogeneous evidence rather than to estimate a pooled treatment effect. Reporting follows the Preferred Reporting Items for Systematic Reviews and Meta-Analyses extension for Scoping Reviews (PRISMA-ScR) (13). No review protocol was registered or published. Consistent with scoping-review methodology, the question and eligibility criteria were structured around population, concept, and context rather than an intervention-comparator framework: patients receiving or considered for radiotherapy; sex- or gender-related differences relevant to safety; and clinical radiation-oncology pathways.

### 2.2 Information source and PubMed search

PubMed was searched on 22 July 2026. It was selected as the principal information source because the review targeted clinically oriented radiation-oncology studies and treatment-related safety outcomes in biomedical literature. The search combined terms for radiotherapy or radiation oncology, sex- or gender-related differences, and patient safety, quality, clinical outcomes, toxicity, adverse events, complications, symptom management, or side effects. No date restriction was applied because safety-relevant sex- and gender-related evidence was expected to be sparse and restricting the search to recent years could have excluded informative earlier observations. The Boolean structure was (radiotherapy terms) AND (sex/gender terms) AND (safety, quality, outcome, or toxicity terms). **Supplementary Material S1** shows the complete query.

### 2.3 Backward citation searching

Reviews and other relevant secondary publications identified during screening were used as contextual sources and as seeds for manual backward reference-list searching. Original reports identified through this route were retrieved and assessed under the same eligibility criteria as the PubMed records. Contextual reviews and other secondary publications were not included as empirical studies and contributed no findings to the Results. The seed reviews leading to the five included citation-derived studies were De Felice et al. (2022; Bjordal), Santaballa Bertrán et al. (2025; Luo), and Borgmann et al. (2009; Bhatia, Pieters, and Palazzi). Their full references and study-level links are provided in **Supplementary Material S1**. This seed-to-study attribution was confirmed retrospectively during revision; the exact search date, individual searcher assignment, reconciliation procedure, and any forward citation searching were not prospectively logged. No additional database search was performed.

### 2.4 Eligibility criteria

Eligibility criteria are summarized in **Table 1**. Safety relevance included care processes and outcomes with a potential implication for preventing, detecting, or managing harm; efficacy outcomes were retained as indirect signals and are presented separately. The ability to attribute an association specifically to radiotherapy (RT) was charted separately and was not an eligibility requirement for prespecified multimodal, survivorship, or RO care-process contexts.

**Table 1:** Eligibility criteria.

Include	Exclude
Studies involving clinical radiotherapy or combination therapy of radiotherapy and systemic or surgical treatments; post-radiotherapy survivorship, any RO care process, or treatment selection in which radiotherapy was an option.	Preclinical or animal research; diagnostic radiation exposure or nuclear medicine
An explicit sex- or gender-related comparison, association, threshold, interaction, or care-process difference with at least one empirical finding relevant to a published patient-safety subcategory	No substantive sex/gender analysis
Findings relevant for patient safety	No empirically mappable safety- or quality-relevant finding
Full-length reports of original clinical research	Reviews, editorials, commentaries, protocols, and conference abstracts.
Full text available in English or German	Full text not available in English or German

### 2.5 Calibration, screening, and study selection

Records were assessed at title/abstract and full-text stages by the four manuscript authors. The reviewer team comprised three radiation oncologists and one medical physicist, all with more than 10 years of professional experience in radiation oncology. For calibration, all four reviewers independently assessed the same shared sample of 15 records from the 159-record PubMed set and subsequently discussed their decisions to align application of the eligibility criteria. One reviewer assessed each remaining record and report. Studies co-authored by a reviewer were screened and extracted exclusively by another team member to avoid a conflict of interest. Uncertain or conflicting classifications were resolved through discussion within the reviewer team. Relevant source passages and derived findings were recorded in a shared extraction table accessible to all four authors; the final study classifications, findings, and mappings were reviewed by the full team. For flow-diagram reporting, each excluded record or report was counted once under one primary reason. The 15 calibration records remained part of the PubMed set and did not constitute an additional identification route.

### 2.6 Sex and gender classification

For each included study, we reviewed the terminology used in the source publication, the variable actually measured, and its reported ascertainment. The focal variable was then classified as sex, gender, both, or unclear. Sex was assigned when the publication identified the variable as sex and its ascertainment was described or otherwise unambiguous. Gender required an operationalized identity, role, relational, or structural measure, while both required the separate operationalization of sex and gender. A binary female/male variable labeled as gender without a defined construct or ascertainment was classified as unclear. Initial classifications were made by the reviewer responsible for the study. Ambiguous cases were resolved through discussion within the full reviewer team.

### 2.7 Patient-safety framework mapping

Findings were mapped deductively to an RO patient-safety framework comprising four categories and 27 subcategories. The four-category, 27-subcategory framework was published in the PaSaGeRO systematic review; the subsequent PaSaRO multi-method study further developed the related questionnaire and evaluated its content validity (1, 2). The reviewer responsible for each study charted the measured variable, treatment context, endpoint, result, and radiotherapy-attribution limitation before mapping. **Figure 1** and **Supplementary Material S2** list all 27 subcategories; working operational boundaries and finding-level mapping rationales are provided in **Supplementary Material S2**. The structure-process-outcome model was additionally used to distinguish direct evaluations of care processes from indirect clinical or technical outcome signals (55, 56). Mapping uncertainties were resolved through discussion within the reviewer team. A finding was a distinct empirical statement about an endpoint in its clinical context, assigned to one primary subcategory. A study could contribute several findings; correlated results within one recorded unit were counted once. Mixed findings varied by endpoint, time point or direction within that unit. Non-significant findings were not interpreted as equivalence. Outcome associations were linked to the care step they could inform, without implying process failure or preventability. Mapping by one reviewer with team discussion was not independently duplicated.

**Figure 1:**
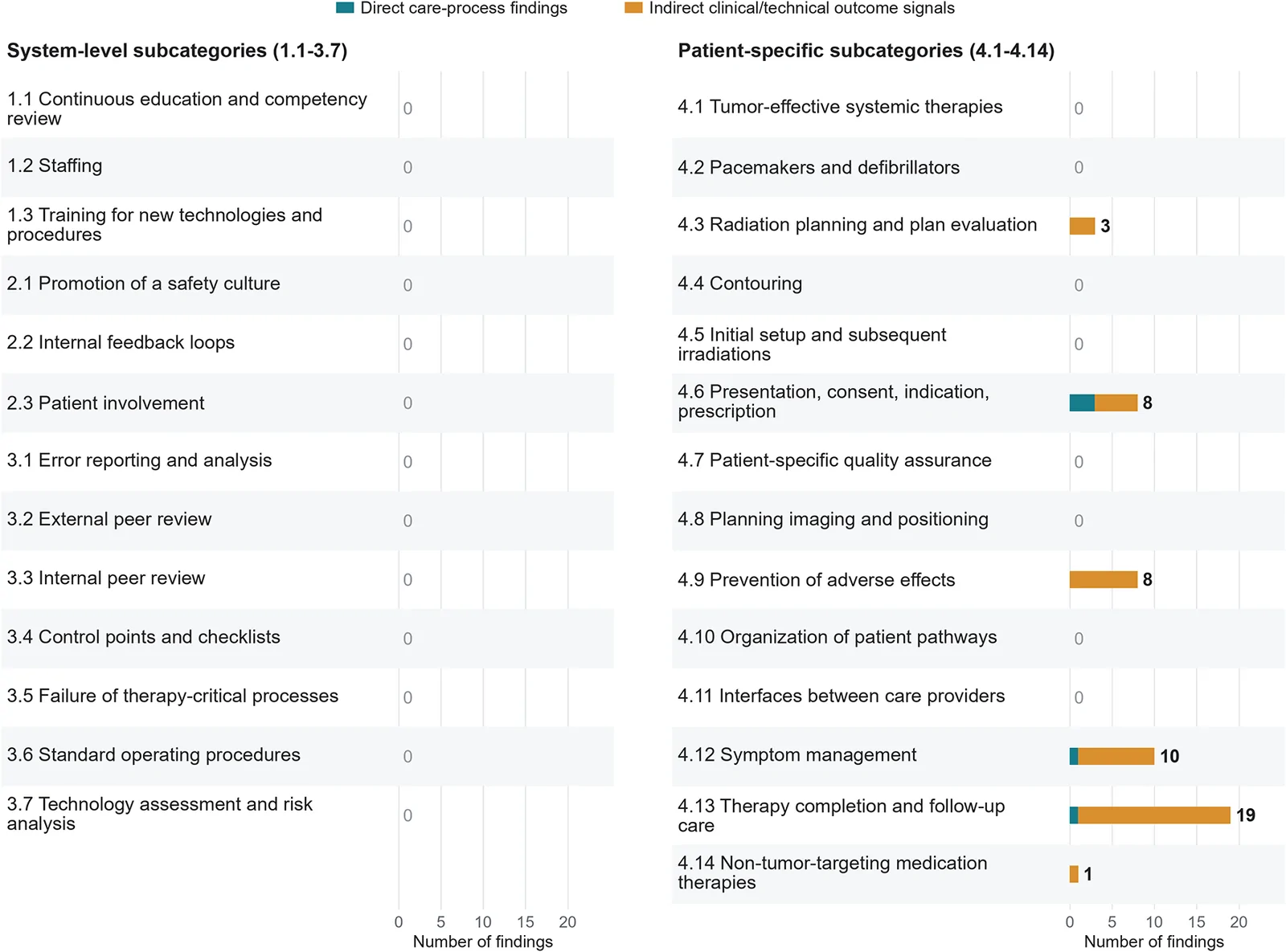
Distribution of 49 findings across the 27 patient-safety subcategories, distinguishing direct care-process findings from indirect clinical or technical outcome signals.

Our working boundaries for the six populated subcategories were: dosimetry and plan evaluation (4.3); presentation, consent, indication and treatment selection (4.6); adverse effects with a potential prevention implication (4.9); physical or psychosocial symptom recognition, monitoring and treatment (4.12); completion, rehabilitation and follow-up, including prognosis as an indirect signal (4.13); and non-tumor medication, complementary-product use and interaction risk (4.14). These are review-specific operational boundaries, not additional published definitions.

### 2.8 Synthesis and critical appraisal

Study characteristics were summarized descriptively. Empirical findings were synthesized narratively, considering their clinical context. Counts describe the distribution of retrieved evidence, not the strength or clinical importance of the evidence. No formal risk-of-bias assessment or certainty-of-evidence grading was performed. Study design, potential confounding, treatment heterogeneity, radiotherapy attribution, and sex/gender terminology were used as narrative qualifiers. The completed PRISMA-ScR checklist is provided as **Supplementary Material S3**.

## 3 Results

### 3.1 Study selection

The PubMed search identified 159 records; none were removed as duplicates or by automation. After 118 title/abstract exclusions, 41 full-text reports were retrieved and assessed for eligibility. Nine were excluded: four lacked safety or quality relevance, three lacked an eligible radiation-oncology context, and two were not available in English or German. This left 32 database-identified studies. Backward citation searching identified six reports; all were retrieved and assessed, one conference abstract-only report was excluded, and five original studies were included. The review therefore comprised 37 primary studies (**Figure 2**).

**Figure 2:**
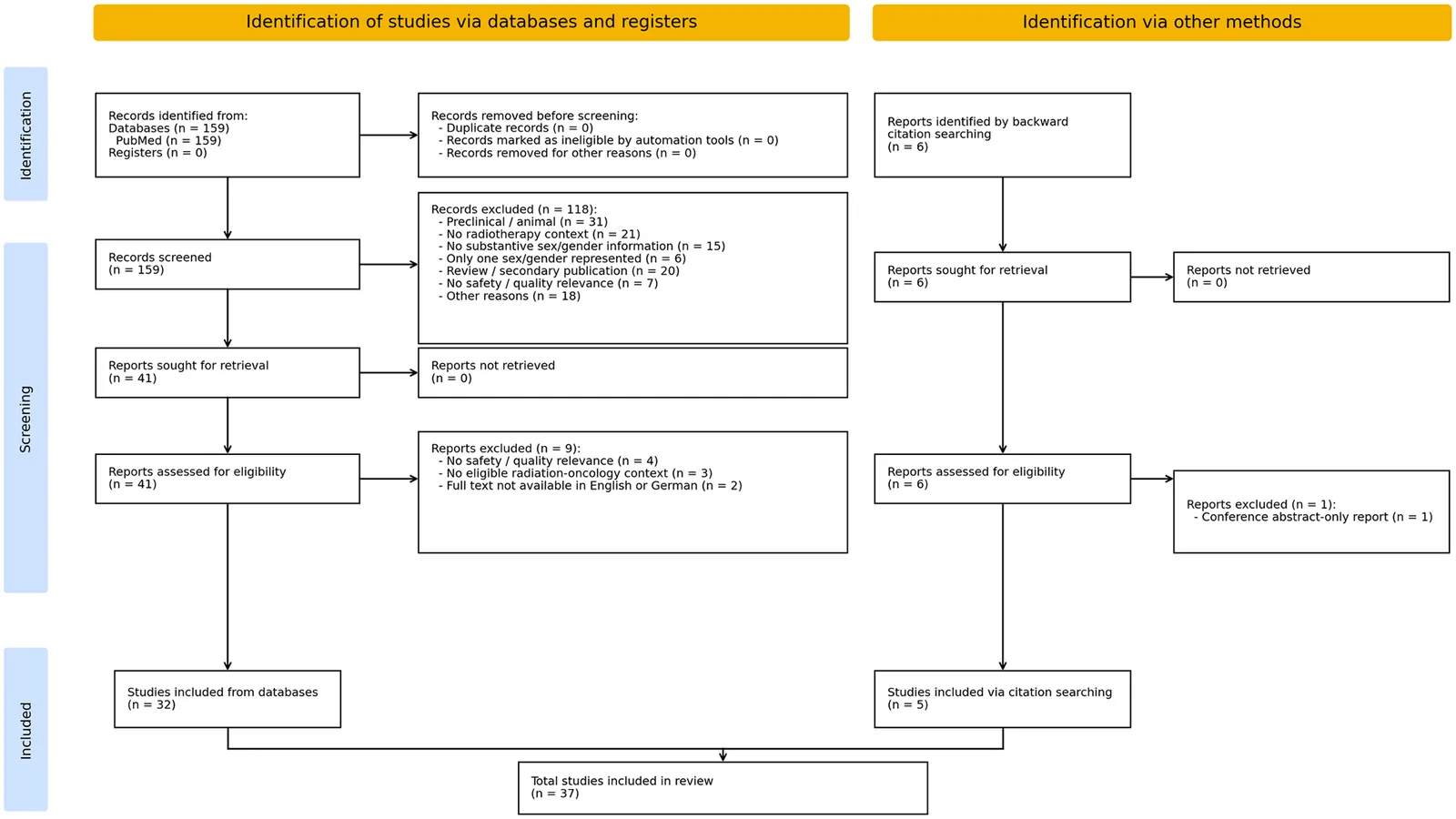
PRISMA-ScR flow diagram of study identification and selection, resulting in 37 studies included from PubMed searching and backward citation searching.

### 3.2 Study characteristics

The 37 studies were published between 1990 and 2026 and included sample sizes from 34 to 11, 774 participants. Most were observational cohorts or registry studies; the evidence also included cross-sectional and descriptive studies, secondary analyses of randomized trials, a prospective registry cohort, a pooled analysis of randomized trials, and a retrospective propensity-score-matched cohort. Seven studies were radiotherapy-specific. Ten involved multimodal treatment in which the contribution of radiotherapy could not be separated, while the remaining studies examined RT-centered multimodal care, mixed treatments, survivorship, historical treatment, treatment selection, supportive-care interventions, or radiation-oncology care processes.

Twenty studies were classified as analyses of sex. Seventeen were classified as unclear because they used inconsistent sex/gender terminology or labeled a binary female/male variable as gender without defining its ascertainment or an operationalized gender construct. No study was classified as gender or both. Characteristics and patient-safety mappings of all included studies are shown in **Table 2**.

**Table 2:** Characteristics, outcomes, adjustment, sex/gender classifications, and patient-safety mappings of the 37 included studies.

Study	Study design	n	Sex/gender class	Radiotherapy context	Framework codes	Outcome and direction	Adjustment of sex-related result
Waber (1990) (31)	Retrospective cohort	66	Sex	Multimodal; RT effect not separable	4.13	Greater cognitive, growth and weight effects in girls	Unclear; adjusted sex effect not established
Mauch (1996) (35)	Retrospective cohort	794	Sex	Historical multimodal treatment	4.13	Sex- and age-dependent second-malignancy patterns	Age/sex-standardized population comparisons
Hertzberg (1997) (30)	Retrospective multicenter study	118	Sex	Multimodal; RT effect not separable	4.13	CNS morphology: no significant sex association	Unclear; adjusted sex effect not established
Bjordal (2001) (37)	Prospective cohort	249	Sex	Mixed treatment/survivorship	4.13	QoL and symptom trajectories differed by endpoint/time	Unclear; adjusted sex effect not established
Bhatia (2002) (36)	Retrospective cohort	47	Sex	RT-specific	4.13	Cataracts more frequent in men	Unclear; adjusted sex effect not established
Baumann (2006) (49)	Retrospective chart review and QoL survey	34	Unclear	Mixed treatment/survivorship	4.13	QoL differences; background-population confounding	Multiplicity correction; confounder adjustment unclear
Pieters (2006) (15)	Retrospective cohort	53	Unclear	RT-specific	4.3	Modelled tolerance higher in women	Yes; multivariable dose–neurotoxicity model
Kaminski (2007) (47)	Prospective cohort	84	Unclear	Multimodal; RT effect not separable	4.9	Anemia, diarrhea and skin toxicity higher in women	Unclear; adjusted sex effect not established
Myrehaug (2008) (29)	Retrospective cohort	615	Sex	Multimodal; RT effect not separable	4.13	Cardiac morbidity higher in men	Yes; age, treatment and cardiac risk factors
Palazzi (2008) (28)	Prospective observational cohort	149	Unclear	RT-centered multimodal	4.12	Pain worse in women	Yes; multivariable toxicity analysis
Martling (2009) (45)	Retrospective population registry	11, 774	Unclear	Historical treatment selection	4.6, 4.13	RT receipt lower in women; local-control benefit in both	Yes for treatment receipt; endpoint-dependent
Sekine (2009) (48)	Retrospective cohort	204	Unclear	Multimodal; RT effect not separable	4.9, 4.13	Higher response in women; toxicity/survival non-significant	Unclear; adjusted sex effect not established
Buffum (2011) (42)	Prospective descriptive cohort	182	Unclear	RT care entry	4.6	Pain/sleep differences; sex-by-pain interactions	Age considered; endpoint-dependent
Wolff (2011) (25)	Prospective cohort	196	Unclear	Multimodal; RT effect not separable	4.9	Severe organ toxicity concentrated in low-BMI women	Sex/BMI analyses; independent sex effect unclear
Lapid (2013) (46)	Secondary analysis of a randomized trial	131	Unclear	RT-centered supportive-care intervention	4.6, 4.12	Early domain-specific QoL differences; none at week 52	Exploratory subgroups; confounder adjustment unclear
Bodnarchuk (2014) (41)	Prospective cohort	119	Sex	RT care entry	4.6	Higher distress in women	No; group comparisons
Tariq (2014) (50)	Retrospective registry	1, 116	Unclear	RT-centered; attribution limited	4.13	Hypothyroidism more frequent in women	No; univariable analysis
Tonning Olsson (2014) (32)	Retrospective cohort	69	Sex	Mixed treatment context	4.13	Lower IQ in boys; tumor size stronger predictor	Yes; multiple regression, tumor/treatment variables
Chow, SC.23 (2017) (44)	Secondary analysis of a randomized trial	298	Unclear	RT-specific	4.6, 4.12	Similar pain response; selected symptom/QoL differences	Yes; baseline demographics and disease site
Chow, SC.20 (2018) (43)	Secondary analysis of a randomized trial	847	Unclear	RT-specific	4.6, 4.12	Similar pain response; selected QoL differences	Unclear; adjusted sex effect not established
Luo (2019) (34)	Retrospective propensity-score-matched cohort	683	Sex	RT-specific	4.13	PFS/OS longer and recurrence lower in women	Yes; propensity matching and multivariable Cox models
Sheehan (2019) (26)	Prospective registry cohort	116	Sex	RT-specific	4.12	Pain/discomfort worsened more in men	Yes; multivariable linear regression
Schuster (2022) (40)	Pooled prospective cohorts	710	Unclear	Multimodal; RT effect not separable	4.3, 4.12	Dose/body mass higher in women; symptom effects mixed	Endpoint-dependent; adjusted sex effect unclear
Zwart (2022) (22)	Retrospective cohort	193	Sex	Mixed treatment; RT subgroup	4.9	Sex-specific muscle-index cut-offs; low index linked to toxicity	Adjusted low-muscle-index effect, not a sex effect
Muhandiramge (2023) (16)	Retrospective cohort	1, 893	Sex	Mixed oncology treatment	4.6	Treatment-receipt difference absent without sex-specific cancers	Yes; multivariable and sensitivity analyses
Bohm (2024) (27)	Prospective cohort	75	Sex	Mixed treatment/survivorship	4.12, 4.13	Sex differences in symptom trajectories	Unclear; adjusted sex effect not established
Mangesius (2024) (33)	Retrospective cohort	108	Sex	RT-specific	4.13	Observed survival longer in women	Yes; multivariable prognostic model
Nieder (2024) (20)	Retrospective cohort	181	Sex	Multimodal; RT effect not separable	4.9	Toxicity/survival differences non-significant	Unclear; adjusted sex effect not established
Agolli (2025) (18)	Retrospective cohort	309	Sex	RT-centered multimodal	4.9, 4.13	Dermatitis and central nodal failures higher in women	Survival modelled; adjustment of these signals unclear
Frehner (2025) (21)	Pooled analysis of randomized trials	499	Sex	Multimodal; RT effect not separable	4.9, 4.13	EFS/OS longer in women; severe adverse events similar	Yes for survival; multivariable Cox models
Heller (2025) (14)	Retrospective single-center cohort	94	Unclear	RT-centered; attribution limited	4.3, 4.13	Atrial doses higher in men; heart-failure odds higher in women	No for sex–heart-failure association
Käsmann (2025) (39)	Prospective cohort	112	Sex	RT care process	4.14	CAM use and potential interaction exposure higher in women	Unclear; adjusted sex effect not established
Man and Ho (2025) (24)	Cross-sectional survey	114	Unclear	Post-RT/survivorship	4.12	Poorer mental health in men; attenuated with optimism	Yes; sequential models including optimism
Wang (2025) (17)	Retrospective two-center cohort	1118	Sex	Mixed-modality comparison	4.6, 4.13	SRS recurrence higher in women; assignment differed	Unadjusted comparisons; independent sex effect unclear
Besserer (2026) (38)	Retrospective cohort	2727	Unclear	Post-RT/survivorship	4.13	Rehabilitation uptake higher in women (25.1% vs 9.6%)	Unclear for reported sex contrast
Dinapoli (2026) (23)	Retrospective cohort	895	Unclear	RT care process	4.12	Greater distress/support use in women; diagnosis-dependent	Predictor models reported; endpoint-dependent
Sui (2026) (19)	Prospective cohort	329	Sex	Multimodal; RT effect not separable	4.9	Hematologic toxicity more severe in women	Microbiome analyses; adjusted sex effect unclear

Adjustment refers to the reported sex-related result, not study quality. “Unclear” means that an adjusted sex effect could not be established from the available reporting; it does not mean that no adjusted analysis was performed. Adjustment sets varied and may leave residual confounding. Trial randomization does not randomize sex. BMI, body mass index; CAM, complementary and alternative medicine; CNS, central nervous system; EFS, event-free survival; OS, overall survival; PFS, progression-free survival; QoL, quality of life; SRS, stereotactic radiosurgery.

### 3.3 Distribution of empirical findings

The 37 studies contributed 49 reportable empirical findings. Thirty-three described a difference or association, 10 were mixed, and six were statistically non-significant.

Seven findings primarily concerned survival, recurrence, or local control. These are shown separately in **Table 3** as a subset of the 49 findings, leaving 42 other findings. They were retained as indirect signals relevant to prognosis and follow-up, not as evidence of preventable harm; the original mapping and counts were unchanged.

**Table 3:** Efficacy-related findings retained as indirect signals within the original evidence map.

Study	Efficacy endpoint	Reported result
Wang (2025) (17)	SRS recurrence	Higher in women (14% vs 8%)
Agolli (2025) (18)	Central high-dose nodal failure	More frequent in women; overall survival comparable
Frehner (2025) (21)	Event-free and overall survival	Longer in women; multimodal regimens
Mangesius (2024) (33)	Survival after SRS	Longer observed survival in women
Luo (2019) (34)	Progression-free/overall survival and recurrence	Longer survival and fewer recurrences in women
Martling (2009) (45)	Local control with preoperative RT	Benefit in both women and men
Sekine (2009) (48)	Response and survival	Higher response in women; no significant survival difference

These seven findings are a subset of the original 49 findings, all mapped to 4.13, and are not counted again. They are indirect prognostic or follow-up signals; they do not establish preventable harm or benefit from a sex-specific safety process. Other endpoints from these studies remain in their original subcategories in S2.

All 49 findings mapped to patient-specific processes within the published radiation-oncology patient-safety framework. Six of the 27 subcategories were populated: radiation planning and plan evaluation; patient presentation, informed consent, indication, and prescription; prevention of adverse effects; symptom management; therapy completion and follow-up care; and monitoring and adjustment of non-tumor-targeting medication therapies. **Figure 1** shows the distribution across all 27 subcategories.

### 3.4 Radiation planning and plan evaluation

Planning-related evidence came from three clinically distinct settings. In a retrospective single-center cohort of 94 patients with post-radiotherapy cardiac events, men had higher maximum atrial doses, while women had higher unadjusted odds of heart failure; dose mediation was not demonstrated (14). This selected cohort also informed Section 3.7.

A cohort of 53 patients treated with high-dose fractionated radiotherapy to fields encompassing the cauda equina provided a second dose-related signal. Cauda-equina dose and the gender variable were associated with late neurotoxicity. The model developed by Pieters et al. estimated that five-year tolerance doses were higher for women than men. The estimates were based on 13 neurotoxicity events and more than half occurred five or more years after treatment (15).

In 710 patients receiving radiochemotherapy for locally advanced rectal cancer, women had a higher total-body mean dose per unit body mass, but deposited energy and weight-normalized tumor-region dose were comparable. Furthermore, this study also included investigations on tumor regression, residual DNA damage, and treatment outcome, which also were comparable. This cohort also reported longitudinal symptom and laboratory findings, which are presented with treatment-related effects in Section 3.6 (40).

### 3.5 Patient presentation, informed consent, indication, and prescription

Evidence on treatment indication and receipt was available for different entities. A large cohort study with 1, 893 elderly patients with mixed tumor sites indicated that women had higher unadjusted odds of receiving cancer treatment, but the association disappeared after sex-specific cancers were excluded (16). In a population registry of 11, 774 patients with rectal cancer, women were less likely to receive preoperative radiotherapy after adjustment for recorded clinical factors; preoperative radiotherapy nevertheless improved local control in both women and men (45). Among 1, 118 patients treated for vestibular schwannoma, women with very small tumors were assigned to surgery less often than men. The same cohort found a higher recurrence rate among women after stereotactic radiosurgery, linking the treatment-selection observation to the follow-up outcome reported in Section 3.7 (17). None of these studies assessed the appropriateness of individual decisions or the documented decision rationale.

Several studies described sex-associated differences in symptoms at presentation. Women more often reported distress in a screening cohort and, in a separate sleep study, pain and poorer subjective sleep; pain-related objective sleep disruption was observed in men but not women (41, 42). Before palliative radiotherapy or re-irradiation for painful bone metastases, selected baseline symptoms differed between women and men, but analgesic response did not differ substantially (43, 44). A multidisciplinary quality-of-life intervention produced early, domain-specific subgroup differences that were no longer evident at week 52 (46).

### 3.6 Treatment-related adverse effects and symptom management

The evidence mapped to prevention of adverse effects and symptom management overlapped clinically around acute toxicity, pain, psychological burden, and quality of life. These two framework subcategories are therefore presented together here, while their finding-level assignments remain separate in **Table 2** and **Supplementary Material S2**.

Rectal-cancer radiochemotherapy was the most frequently studied treatment setting. During intensified preoperative treatment, all seven grade 4 toxicities occurred in women with a body mass index below 22 kg/m2, whereas hematologic toxicity did not differ significantly by sex (25). A separate prospective cohort of locally advanced rectal cancer found more severe leukopenia, lymphopenia, and neutropenia among women and reported exploratory sex-associated microbiome and arginine-metabolism patterns (19). Another cohort reported more anemia, diarrhea, and skin toxicity among women (47). A longitudinal analysis of 710 patients showed heterogeneous, time-dependent changes: women entered treatment with poorer quality-of-life scores and showed greater deterioration in nausea and vomiting, appetite, and diarrhea, but most symptoms returned toward baseline within ten weeks, most laboratory measures did not clearly differ, and tumor regression and measured radiosensitivity were comparable (40).

In head and neck cancer, findings differed by endpoint and treatment phase. Women treated for locally advanced pharyngeal or laryngeal squamous-cell carcinoma had more grade 3 or higher dermatitis than men (12.7% vs 5.3%) (18). In another cohort, female sex was independently associated with more pain, whereas mucositis, weight loss, and dysphagia were associated mainly with treatment intensification, concomitant chemotherapy, previous surgery, or age (28). In a 193-patient cohort, 135 participants received head and neck (chemo)radiotherapy; sex-specific skeletal muscle index cut-offs were derived, and low muscle index was independently associated with all-cause grade 3 or higher toxicity in this subgroup. This was a finding about sex-specific risk-marker thresholds, not an independent female/male effect (22). A prospective quality-of-life cohort showed different symptom trajectories rather than a uniform direction: during treatment, men reported more pain-related burden and greater need for pain medication or nutritional support, whereas women reported more weight loss and dry mouth; at three months, women reported more dry mouth, weight loss, and sticky saliva (27).

Lung-cancer studies provided several non-significant or mixed findings. In palliative (chemo)radiotherapy for non-small-cell lung cancer, toxicity and survival did not differ materially between women and men (20). In pooled trials of neoadjuvant treatment for resectable stage III non-small-cell lung cancer, grade 3 or higher adverse events and treatment discontinuation were similar, although women had longer event-free and overall survival and lower non-cancer mortality (21). During concurrent chemoradiotherapy for stage III non-small-cell lung cancer, grade 3–4 neutropenia, febrile neutropenia, and severe esophagitis did not differ; a higher response rate among women did not translate into differences in progression-free or overall survival (48).

In a radiation-oncology cohort spanning adult and pediatric diagnoses, women reported more distress, anxiety, depression, and voluntary psychosocial-support use overall, but no female/male difference was found for rectal or anal cancer and psychological burden was not associated with radiotherapy toxicity level (23). Among post-radiotherapy survivors with mixed cancers, men had poorer unadjusted mental-health measures, but associations with depression and stress attenuated after optimism was considered (24). Male sex predicted worsening pain and discomfort after stereotactic radiosurgery for brain metastases (26). In two secondary analyses of palliative bone-metastasis trials, the baseline differences described above did not translate into a female/male difference in analgesic response, although isolated mood, emotional, and other patient-reported changes differed (43, 44). Finally, early quality-of-life responses to a multidisciplinary intervention for advanced cancer varied by subgroup and domain, but no female/male differences remained at week 52 (46).

### 3.7 Therapy completion and follow-up care

Cardiac follow-up findings were linked to different tumor and treatment contexts. The selected thoracic cohort described in Section 3.4 showed an unadjusted heart-failure association (14). Conversely, among Hodgkin lymphoma survivors treated with doxorubicin and mediastinal radiotherapy, male sex, older age, and diabetes were associated with adverse cardiac events (29).

Pediatric late-effect studies showed no consistent direction. In survivors of childhood acute lymphoblastic leukemia, one cohort found no association between sex and morphological central-nervous-system alterations after cranial irradiation and methotrexate-based treatment (30), whereas another historical cohort reported more severe cognitive impairment, short stature, and excessive weight gain among girls (31). In survivors of pediatric brain tumors, lower IQ was associated with male sex, younger age, tumor size, and whole-brain radiotherapy, but tumor size was the stronger predictor (32).

Efficacy findings are distinguished from harm and care-process findings in **Table 3**. Disease-control findings likewise varied by tumor entity. Women had longer observed survival after stereotactic radiosurgery for brain metastases (33) and longer progression-free and overall survival after definitive radiotherapy for esophageal squamous-cell carcinoma, accompanied by fewer locoregional recurrences and distant metastases (34). In the vestibular-schwannoma cohort that also showed a treatment-assignment difference, recurrence after stereotactic radiosurgery was higher among women (14% vs 8%) (17). In locally advanced pharyngeal or laryngeal cancer, the cohort with more severe dermatitis among women also showed more central high-dose nodal failures among women, while overall survival was comparable (18). The lung-cancer studies combined favorable survival among women in pooled neoadjuvant trials with non-significant survival differences in palliative and concurrent-chemoradiotherapy cohorts (20, 21, 48). In rectal cancer, local control improved with preoperative radiotherapy in both women and men despite the lower treatment receipt among women (45).

Other follow-up findings concerned second malignancies, cataract, hypothyroidism, longitudinal quality of life, and rehabilitation. After historical Hodgkin lymphoma treatment, women irradiated at a young age had higher risks of breast and other solid second malignancies, whereas men showed a different pattern that included leukemia and non-Hodgkin lymphoma (35). Among patients treated with curative orbital radiotherapy for primary orbital lymphoma, cataracts were more frequent in men, but cataract formation was not associated with dose, fraction size, beam arrangement, steroid use, or lens blocking (36). Head and neck cohorts showed female/male differences in longitudinal symptoms and quality of life, including emotional function, sleep, dry mouth, sensory symptoms, weight loss, and nutritional support; the direction depended on treatment phase and endpoint (27, 37, 49). Hypothyroidism after external-beam radiotherapy for head and neck cancer was more frequent among women (50). One study evaluated a follow-up process directly: despite standardized information and administrative support, rehabilitation was initiated by 25.1% of source-defined women and 9.6% of men after radiotherapy (38).

### 3.8 Monitoring and adjustment of non-tumor-targeting medication therapies

Medication-related evidence came from a prospective survey of 112 radiotherapy patients with different tumor entities. Women reported more complementary-medicine use and more frequent exposure to products with possible or known interaction potential whereas interest in counseling did not differ. The study assessed potential exposure but did not measure actual interactions or adverse drug events (39).

## 4 Discussion

### 4.1 Principal findings

The retrieved literature contained sex-related signals with potential relevance to the safe design and delivery of radiotherapy, but the evidence remained concentrated in six patient-specific subcategories and was dominated by clinical or technical outcomes whereas direct evaluations of care processes were uncommon. No study operationalized multidimensional gender. The direction and apparent relevance of the reported differences depended on diagnosis, treatment, endpoint, and time point rather than indicating consistent vulnerability of women or men.

This pattern is consistent with earlier reviews that characterized radiotherapy evidence as heterogeneous and insufficient to estimate a general sex-dependent treatment effect (4, 10, 54). The present review adds a patient-safety perspective. The retrieved evidence clustered around toxicity, symptoms, dosimetry, and disease outcomes, whereas direct evidence on care processes, communication, incident prevention, and sex- or gender-informed interventions was sparse. The framework distinguishes outcome associations from actionable care processes and makes unstudied organizational and system-level areas visible; an outcome association alone does not demonstrate preventability or an effective process change (1, 2).

### 4.2 Findings in relation to existing literature

Several studies reported female–male differences in treatment-related toxicity, but the direction and magnitude varied by tumor site, treatment regimen, and endpoint. Greater acute side effects among women were reported in head and neck and rectal-cancer chemoradiotherapy cohorts (18, 19, 25, 47), and the longitudinal rectal-cancer analysis also showed worse transient symptom and laboratory patterns among women (40). By contrast, toxicity was similar in palliative and neoadjuvant non-small-cell lung cancer cohorts and in stage III concurrent chemoradiotherapy (20, 21, 48). The low-muscle-mass study points to body composition as a candidate risk marker, but its internally derived cut-offs had limited discrimination and no external validation (22). Body composition may be an explanatory or predictive variable rather than a mechanism attributable to sex itself, and any proposed threshold requires independent validation. These findings refine the broader signal described in prior oncology reviews (4): anatomy, dose-volume exposure, systemic therapy, body composition, and baseline risk should be modeled together before sex is interpreted as an independent predictor. Future cohorts should prespecify sex-by-treatment and sex-by-dose interactions and test whether sex adds predictive value to dose-response models after established clinical and dosimetric predictors are included. Systemic therapy may confound, mediate, or modify observed associations with toxicity; future studies should therefore capture component-specific treatment exposure and test whether sex adds information beyond anatomy, dose, body composition, comorbidity, tumor factors, and systemic therapy.

Symptoms and quality of life were similarly context- and time-dependent. Distress, mental-health measures, pain, sleep, head and neck symptoms, and treatment-related quality of life differed in opposing directions across populations and endpoints (23, 24, 26–28, 40–42, 47, 49). The two palliative bone-metastasis analyses are particularly informative: individual baseline symptoms and some emotional or mood changes differed, but radiotherapy response did not (43, 44). This supports universal longitudinal patient-reported outcome collection, with subgroup interactions prespecified and interpreted alongside diagnosis, symptom burden, analgesic use, and treatment phase. A broader, partly overlapping head and neck review likewise characterized the evidence as sparse and inconsistent (8). More intensive subgroup-specific screening or referral should therefore be tested in interventions rather than inferred from observational differences.

The pediatric studies assessed clinically different late effects and should be interpreted separately. One historical cohort reported more severe cognitive, growth, and weight effects among girls treated for childhood acute lymphoblastic leukemia, whereas a pediatric brain-tumor cohort associated lower IQ with male sex and found tumor size to be a stronger predictor (31, 32). A further childhood acute lymphoblastic leukemia cohort found no sex association with morphological central nervous system alterations (30). The studies differed in disease, historical treatment, developmental age, imaging and neuropsychological outcomes, and covariate structure. Reviews of cranial irradiation provide biological plausibility for sex-associated neurocognitive and endocrine differences, but several mechanisms remain unresolved (51, 52). Exposure-based follow-up remains clinically coherent, while future cohorts should test whether sex modifies specific dose-response relationships after accounting for age, tumor site and size, surgery, chemotherapy, and endocrine status.

Several studies investigated sex-related differences in thoracic dose distributions or reported cardiac long-term outcomes. The selected thoracic cohort provided an unadjusted heart-failure association; selection, tumor-site case mix and systemic therapy limit its interpretation, and dose mediation was not established (14). A Hodgkin lymphoma survivorship cohort, by contrast, associated male sex, older age, and diabetes with adverse cardiac events in a group where radiotherapy was combined with doxorubicin (29). A cardio-oncology review that included this cohort also found no uniform direction and emphasized interactions between treatment exposure, baseline cardiovascular risk, and sex-related factors (5). In thoracic malignancies, radiotherapy is commonly combined with systemic agents, making the attribution of late effects to an individual treatment component difficult. This challenge is particularly relevant when comparing lymphoma treatment concepts, in which cardiac and second-malignancy risks depend on age, baseline risk, systemic therapy, and dose distribution. A modern planning study in primary mediastinal B-cell lymphoma illustrates how alternative techniques can be compared using organ doses and modeled toxicity risks, but such modeling does not establish a sex-specific mechanism or constraint (57). Likewise, in our review, one Hodgkin cohort described an increased risk of breast and other solid second malignancies among young women and different second-malignancy patterns among men (35).

Only limited data are available addressing possible sex or gender differences in treatment availability or choice of treatment. An apparent female advantage in overall cancer-treatment receipt disappeared after sex-specific cancers were removed (16), while women in a historical rectal-cancer registry received preoperative radiotherapy less often despite benefit in both groups (45). Neither finding establishes inequitable or inappropriate care. Audits might compare clinically similar cases and capture anatomy, comorbidity, available options, patient preference, indication, and documented decision rationale before targeted measures are designed.

Few studies looked deeper into specific planning aspects. The higher maximum atrial doses among men in the thoracic cohort were not linked to a demonstrated sex-by-dose mechanism (14). Model-derived cauda-equina tolerance estimates arose from only 13 neurotoxicity events and require long-latency replication (15). In rectal-cancer radiochemotherapy, body-mass-normalized total-body dose was higher among women, while tumor-region dose, residual DNA damage, tumor regression, and outcome did not differ (40). Prior reviews similarly proposed possible sex-dependent radiation effects but could not quantify them, and none of the included studies tested or validated sex- or gender-specific planning constraints for most organs at risk (4, 10). Pediatric Hodgkin protocols provide a more specific precedent because treatment decisions addressed defined gonadal and breast-cancer risks, although long-term benefits remained incompletely resolved (53). Future work should model anatomy and dose-volume exposure directly, test sex-by-dose interactions, and validate any planning rule against both toxicity and tumor control.

### 4.3 Patient-safety implications

The included studies identify points in the radiotherapy care pathway at which sex-stratified evaluation may be informative. Higher symptom burden among women at treatment initiation and divergent trajectories during treatment suggest that standardized longitudinal screening should be evaluated with prespecified subgroup analyses rather than restricted *a priori* to one sex. The palliative-radiotherapy studies suggest that treatment effectiveness may not differ substantially by sex in the investigated settings; however, they provide no evidence on whether access to palliative radiotherapy was equitable (43, 44). Rehabilitation initiation also differed markedly despite standardized information and administrative support (38). This finding supports prospective evaluation of whether standardized proactive outreach reduces the observed difference. Findings on radiation dose distribution in the thoracic and pelvic cohorts point toward possible sex-related variation in organ-at-risk dosimetry, but no included study tested or validated sex-specific planning constraints. Individual variability in organ-substructure sensitivity, competing contributors to outcomes such as cardiac mortality, and general interindividual sensitivity would need to be accounted for before such constraints could be considered, and prospective validation against both toxicity and tumor control would be required.

For current practice, the findings support accurate documentation of sex and gender variables, universal risk-adapted symptom monitoring, routine medication reconciliation including complementary products, and individualized planning based on anatomy and dose rather than sex alone. The evidence does to date not support routine sex-specific thresholds or pathways; such approaches require prospective validation against both safety and tumor-control outcomes.

### 4.4 Evidence gaps and research priorities

The studies examined treatment receipt or assignment, rehabilitation initiation, and a supportive-care intervention, but none established that a sex- or gender-targeted pathway improves patient outcomes. An observed difference can identify where prevention, monitoring, decision quality, or follow-up should be studied. For example, women more often reported complementary-medicine products with possible or known interaction potential (39). A structured history that asks every patient about medication and complementary products, and is repeated when treatment changes, is an example measurable safety process. Whether an additional interview or counseling pathway should be directed at particular subgroups must be tested prospectively.

Research in the populated subcategories should test whether sex adds predictive value to condition-specific dose-response and toxicity models after anatomy, systemic therapy, baseline risk, body composition, comorbidity, and tumor biology are included. Symptom signals require trials of defined assessment and referral processes, with detection, completed referral, symptom burden, and patient-reported benefit as outcomes. Studies of treatment, follow-up, and rehabilitation pathways should collect clinical, preference, communication, access, and social data. Multidimensional gender needs to be measured directly rather than inferred from an administrative female/male field.

The absence of retrieved evidence in 21 subcategories should not be interpreted as evidence that sex or gender is irrelevant in these domains. It may reflect both the current literature and the scope of the targeted search strategy. Searches tailored to health services, nursing, implementation, qualitative research, incident learning, and gray literature may identify evidence not retrieved by the outcome-oriented PubMed query. Subsequent systems studies could examine whether near misses, delays, communication failures, incident patterns, or the performance of safety processes differ according to clearly defined sex- or gender-related variables. **Table 4** summarizes research priorities derived from the mapped evidence and these gaps.

**Table 4:** Research priorities derived from the mapped evidence and identified gaps.

Priority	Exemplary research question
Prediction of treatment-related toxicity	Does sex or gender add predictive value beyond anatomy, dose-volume exposure, body composition, systemic therapy, comorbidity, and tumor factors?
Symptom monitoring and supportive care	Do standardized longitudinal assessments, treatments and predefined referral pathways improve detection and management across sex and gender groups?
Treatment, follow-up, and rehabilitation pathways	Are observed differences explained by clinical indication, patient preference, communication, access, or process design, and do standardized pathways reduce unwarranted variation? Can we identify optimized treatment pathways stratified by sex or gender?
Medication and complementary-product safety	Does structured and repeated medication reconciliation identify interaction risks and prevent adverse events?
Measurement and reporting of sex and gender	Does consistent ascertainment and reporting improve interpretability and enable valid subgroup analyses?

The focus on female/male comparisons does not cover all sex-specific or gender-diverse safety processes; empty framework categories therefore also reflect the scope of this review.

### 4.5 Sex and gender terminology

The contrast between 20 studies classified as sex and 17 classified as unclear shows why source terminology, the measured variable, and the review classification must remain separate. As shown in **Table 2**, the unclear group included studies that used the term gender for an unascertained binary female/male variable or did not report how the variable was defined (14, 15, 23–25, 28, 38, 40, 42–50). Such data permit category-associated descriptions but do not distinguish biological sex effects from social gender mechanisms. We therefore followed the SAGER recommendation to report definitions, ascertainment, analysis, and interpretation transparently (3).

Future RO studies should use consistent reporting: state whether the variable represents sex or gender and how it was ascertained; define all categories and missing data; provide sex-disaggregated descriptive and outcome data; prespecify and justify interaction analyses; adjust for anatomy, dose, systemic therapy, body composition, tumor characteristics, and relevant social factors; and avoid attributing a difference to biological or social mechanisms that were not measured. Better documentation of sex and gender is essential for judging whether reported differences are clinically meaningful and reproducible. Sex and gender should also be considered alongside intersecting characteristics such as age, comorbidity, tumor site, socioeconomic conditions, and access to care rather than interpreted in isolation.

### 4.6 Limitations

First, this review used a targeted PubMed search supplemented by manual backward citation searching. PubMed was selected for its coverage of clinically oriented biomedical and radiation-oncology literature, but restriction to one bibliographic database and a phrase-dependent query may have missed organizational, qualitative, implementation, nursing, psychosocial, and gray literature. An unpopulated subcategory therefore indicates that no evidence was retrieved by this strategy, not evidence that sex or gender is irrelevant to that domain. Five of the 37 studies were identified only through citation searching, and studies reporting sex solely as a covariate may have been captured selectively. The seed-to-study links were confirmed retrospectively, but the backward-search date, individual searcher assignment and reconciliation procedure were not prospectively logged. Full-text assessment was limited to English and German.

Second, the independent 15-record calibration aligned application of the eligibility criteria, whereas the remaining screening, data charting and mapping were not independently duplicated. Relevant source passages and findings were documented in a shared table and reviewed by the team; this does not replace independent duplicate mapping.

Third, mapping findings to the framework required reviewer judgment, and the resulting counts describe the distribution rather than the strength or clinical importance of the evidence. No formal risk-of-bias or certainty assessment was conducted. Most studies were observational, treatment settings and outcomes were heterogeneous, and adjustment for confounding varied. Only seven studies were radiotherapy-specific; radiotherapy could not be separated from other treatment in 10 multimodal studies. Binary and poorly defined source variables further limited causal interpretation of sex and precluded conclusions about multidimensional gender.

## 5 Conclusions

The available literature indicates that sex can be relevant to patient safety in radiation oncology, while multidimensional gender remains essentially unstudied.

Differences in complementary-medicine use, rehabilitation uptake and psychosocial-support use may also reflect gender-related roles, behavior or access to care; these mechanisms were not directly measured and cannot be inferred from a binary sex variable.

The evidence was concentrated in clinical or technical outcomes and varied by diagnosis, treatment, endpoint, and time point. It identifies plausible safety-relevant signals in treatment planning, toxicity, symptom management, treatment pathways, medication use, and follow-up, but no included study established that an adapted sex- or gender-informed process improves patient safety. Future studies should evaluate defined care processes prospectively, document sex and gender consistently, and determine whether these variables add predictive and clinical value beyond anatomy, dose, systemic therapy, body composition, tumor factors, and social context.

## Data Availability

The full study-level evidence matrix, category mapping, complete search strategy, and completed PRISMA-ScR checklist are provided as **Supplementary Materials S1–S3**.

## Footnotes

1 World Health Organization. Patient safety. 11 September 2023. https://www.who.int/news-room/fact-sheets/detail/patient-safety (accessed 1 October 2026).

## Supplementary material

Supplementary Material S1 PubMed query executed on 22 July 2026 and retrospectively confirmed seed reviews for backward citation searching. The query is presented in the normalized form reported by PubMed Query Translation; spacing differences from the submitted string did not alter the retrieved set. No date restriction or additional interface filter was applied.

Supplementary Material S2 Full study-finding-category matrix and complete 27-subcategory evidence map, including working operational boundaries, source terminology, measured variable, review classification, finding-level mapping rationale, narrative use, and limitations on radiotherapy-specific attribution.

Supplementary Material S3 Completed PRISMA-ScR checklist.
