# Supplementary material for: Sex- and gender-related aspects of patient safety in radiation oncology: a scoping review
Source: Front Oncol. 2026 Oct 27;16:1962214. doi: 10.3389/fonc.2026.1962214 (PMC13749819; doi:10.3389/fonc.2026.1962214)
Supplement: Supplementary file 1 [file Table1.docx]

**Supplementary Tables S1-S2**

**Supplementary Table S1. PubMed search strategy**

*Final query executed on 22 July 2026. The query is presented in the normalized form reported by PubMed Query Translation; spacing differences from the submitted string did not alter the retrieved set; no date restriction or additional interface filter was applied.*

| **Search element** | **Reported information** |
| --- | --- |
| **Information source** | PubMed |
| **Provider/platform** | US National Library of Medicine, PubMed |
| **Search date** | 22 July 2026 |
| **Records retrieved** | 159 |
| **Date restriction** | None |
| **Additional interface filters** | None recorded |
| **Search strategy** | ("radiotherapy"[Title/Abstract] OR "radiation therapy"[Title/Abstract] OR "radiation oncology"[Title/Abstract] OR "radiation treatment"[Title/Abstract] OR "irradiation"[Title/Abstract] OR " radiosurgery"[Title/Abstract] OR "SBRT"[Title/Abstract] OR "IMRT" OR "EBRT"[Title/Abstract]) AND ("sex differences"[Title/Abstract] OR "sex-related"[Title/Abstract] OR "sex-specific"[Title/Abstract] OR "gender-specific"[Title/Abstract] OR "gender-related"[Title/Abstract] OR "gender medicine"[Title/Abstract] OR "gender-sensitive care"[Title/Abstract] OR "gender bias"[Title/Abstract] OR "sex factors"[Title/Abstract] OR "gender factors"[Title/Abstract] OR "gender"[Title] OR "sex"[Title]) AND ("patient safety"[Title/Abstract] OR "quality"[Title/Abstract] OR "treatment outcome"[Title/Abstract] OR "clinical outcome"[Title/Abstract] OR "toxicity"[Title/Abstract] OR "adverse events"[Title/Abstract] OR "complications"[Title/Abstract] OR "symptom management " OR "side effects"[Title/Abstract]) |

## Supplementary Table S1B. Seed reviews for the five included citation-derived studies

Seed-to-study attribution was confirmed retrospectively during revision. This reconstruction does not supply a prospectively recorded search date, searcher assignment, or reconciliation log. The table identifies the seeds for the five included studies; it is not a claim that these were the only secondary sources consulted.

| **Seed review** | **Included original study** | **Original-study DOI** |
| --- | --- | --- |
| De Felice F, Locati LD, Ronchi S, Thariat J, Orlandi E. Quality of life and financial toxicity after (chemo)radiation therapy in head and neck cancer: are there any sex- or gender-related differences? Tumori. 2022;108:522–525. https://doi.org/10.1177/03008916221078885 | Bjordal et al. (2001) [37] | https://doi.org/10.1097/00005537-200108000-00022 |
| Santaballa Bertrán A, et al. Sex-related differences in the efficacy and toxicity of cancer treatments. Clin Transl Oncol. 2025;27:3636–3646. https://doi.org/10.1007/s12094-025-03893-2 | Luo et al. (2019) [34] | https://doi.org/10.1186/s13014-019-1278-0 |
| Borgmann K, Dikomey E, Petersen C, Feyer P, Hoeller U. Sex-specific aspects of tumor therapy. Radiat Environ Biophys. 2009;48:115–124. https://doi.org/10.1007/s00411-009-0216-1 | Bhatia et al. (2002) [36] Pieters et al. (2006) [15] Palazzi et al. (2008) [28] | https://doi.org/10.1016/S0360-3016(02)02966-8 https://doi.org/10.1016/j.ijrobp.2005.04.019 https://doi.org/10.1016/j.ijrobp.2007.06.022 |

**Supplementary Table S2A. Empirical study-finding-category matrix**

The unit is one reportable empirical finding mapped to one primary patient-safety subcategory. Seven efficacy-related findings, identified in main-text Table 3, are retained within the original 49 rows. The 49 rows represent 37 studies; a study may contribute more than one distinct finding. Additional PS code(s) identify other empirical subcategory mappings contributed by the same study and refer to separate findings. Reference numbers correspond to the main manuscript; no independent supplementary reference sequence is used.

| **Study [ref]** | **Design; n** | **Sex/gender classification** | **RT context / attribution** | **Primary patient-safety mapping** | **Empirical finding** | **Patient-safety relevance** | **Additional PS code(s)** |
| --- | --- | --- | --- | --- | --- | --- | --- |
| Heller (2025) [14] | Retrospective single-center cohort; n = 94 | Unclear | RT-centered; attribution limited. Thoracic RT was central, but the heart-failure association was not shown to be dose-mediated and adjustment for cardiotoxic systemic therapy was limited. | 4.3 Radiation planning and plan evaluation (Patient-specific Processes; Outcome) | Men received higher maximum atrial doses. Pericardial disease was associated with maximum dose to the pericardium, left atrium, and whole heart; sex-specific planning practice or constraints were not evaluated. | Higher atrial doses in men and dose-associated pericardial disease indicate that cardiac substructure exposure should be evaluated by sex, although sex-specific planning thresholds were not tested. | 4.13 |
| Pieters (2006) [15] | Retrospective cohort; n = 53 | Unclear | RT-specific. The finding directly concerns cauda equina dose tolerance after fractionated irradiation. | 4.3 Radiation planning and plan evaluation (Patient-specific Processes; Outcome) | Cauda equina dose and reported gender were associated with neurotoxicity, with higher estimated tolerance doses in women than men. | The observed difference in cauda equina neurotoxicity suggests that sex may modify dose tolerance, but the evidence is insufficient to define sex-specific constraints. |  |
| Schuster (2022) [40] | Prospective cohort; n = 710 | Unclear | Multimodal; RT effect not separable. Patients received radiochemotherapy; radiation and systemic-treatment effects cannot be separated. | 4.3 Radiation planning and plan evaluation (Patient-specific Processes; Outcome) | Body-size-related whole-body dose differed, but the measured tumor-region exposure and biological or clinical response did not support a general difference in tumor radiosensitivity. | Body-size-related whole-body dose differences support checking dose optimization across sex-related anthropometric variation; no general difference in tumor radiosensitivity was demonstrated. | 4.12 |
| Muhandiramge (2023) [16] | Retrospective cohort; n = 1893 | Sex | Mixed oncology treatment. Treatment receipt was studied across oncology modalities; a radiotherapy-specific effect is not isolated. | 4.6 Patient presentation, informed consent, indication and prescription (Patient-specific Processes; Process) | An apparent female advantage in receipt of cancer treatment disappeared after sex-specific cancers were excluded. | After sex-specific cancers were excluded, treatment receipt did not differ, providing no evidence of a sex-related access disparity in this cohort. |  |
| Wang (2025) [17] | Retrospective two-center cohort; n = 1118 | Sex | Mixed-modality comparison. The study compares surgery and stereotactic radiosurgery; selection and outcome effects may interact. | 4.6 Patient presentation, informed consent, indication and prescription (Patient-specific Processes; Process) | Women with very small tumors were less often assigned to surgery than men; the recurrence outcome is charted separately. | Sex-associated modality selection for very small tumors warrants audit for equitable decision-making, but the study did not determine whether the difference represented under- or overtreatment. | 4.13 |
| Bodnarchuk (2014) [41] | Prospective cohort; n = 119 | Sex | RT care process. Distress was assessed at entry to radiotherapy; it was not an effect of delivered radiation. | 4.6 Patient presentation, informed consent, indication and prescription (Patient-specific Processes; Outcome) | Women reported a greater burden of selected symptoms at entry to radiotherapy. | Greater symptom burden among women at radiotherapy entry supports sex-stratified baseline assessment so that pre-existing needs are not misattributed to treatment. |  |
| Buffum (2011) [42] | Prospective cohort; n = 182 | Unclear | RT care process. Pain and sleep were measured at radiotherapy initiation rather than as treatment effects. | 4.6 Patient presentation, informed consent, indication and prescription (Patient-specific Processes; Outcome) | Pain and sleep differences varied by subjective versus objective measure and by subgroup. | Discordant subjective and objective pain and sleep findings caution against relying on a single assessment method and support sex-stratified symptom evaluation. |  |
| Chow (2018) [43] | Secondary analysis of a randomized trial; n = 847 | Unclear | RT-specific. Palliative re-irradiation was randomized, but subgroup analyses were secondary. | 4.6 Patient presentation, informed consent, indication and prescription (Patient-specific Processes; Outcome) | Selected baseline symptoms differed before palliative re-irradiation. | Baseline symptom differences before re-irradiation should be considered in treatment selection, counseling, and interpretation of subsequent toxicity or response. | 4.12 |
| Chow (2017) [44] | Secondary analysis of a randomized trial; n = 298 | Unclear | RT-specific. All patients received palliative radiotherapy; sex/gender analyses were secondary and not randomized. | 4.6 Patient presentation, informed consent, indication and prescription (Patient-specific Processes; Outcome) | Selected baseline symptoms differed before palliative radiotherapy, although most characteristics were comparable. | Baseline symptom differences before palliative radiotherapy support sex-stratified assessment when counseling patients and interpreting subsequent outcomes. | 4.12 |
| Martling (2009) [45] | Retrospective cohort; n = 11774 | Unclear | Historical treatment selection. Treatment receipt and outcomes reflect historical registry practice and residual confounding. | 4.6 Patient presentation, informed consent, indication and prescription (Patient-specific Processes; Process) | A historical registry showed a sex/gender-labeled difference in treatment receipt, without establishing inappropriate care. | A historical difference in treatment receipt is a potential equity signal, but appropriateness cannot be judged without indication and case-mix data. | 4.13 |
| Lapid (2013) [46] | Secondary analysis of a randomized trial; n = 131 | Unclear | Mixed oncology treatment. The supportive-care intervention was not specific to radiotherapy and subgroup analyses were exploratory. | 4.6 Patient presentation, informed consent, indication and prescription (Patient-specific Processes; Outcome) | A baseline quality-of-life imbalance limited interpretation of subsequent subgroup differences. | Baseline quality-of-life imbalance can confound subgroup outcome comparisons and should be accounted for when counseling patients and evaluating treatment benefit. | 4.12 |
| Agolli (2025) [18] | Retrospective cohort; n = 309 | Sex | RT-centered multimodal. Definitive/adjuvant radiotherapy was central, but systemic treatment and disease factors may contribute. | 4.9 Prevention of adverse effects (Patient-specific Processes; Outcome) | Women had a higher rate of grade 3 or higher dermatitis than men during definitive or adjuvant radiotherapy (12.7% vs 5.3%; p=0.037). | Higher severe dermatitis rates in women support sex-stratified skin-toxicity surveillance; no sex-specific preventive regimen was tested. | 4.13 |
| Sui (2026) [19] | Prospective cohort; n = 329 | Sex | Multimodal; RT effect not separable. Hematologic toxicity occurred during neoadjuvant chemoradiotherapy. | 4.9 Prevention of adverse effects (Patient-specific Processes; Outcome) | Women experienced more severe hematologic adverse events during neoadjuvant chemoradiotherapy. Microbiome associations were exploratory and do not establish a causal or clinically validated intervention target. | Higher hematologic toxicity in women supports closer blood-count monitoring during chemoradiotherapy; microbiome findings remain exploratory and do not justify a targeted intervention. |  |
| Nieder (2024) [20] | Retrospective cohort; n = 181 | Sex | Multimodal; RT effect not separable. The cohort includes palliative radiotherapy and chemoradiotherapy. | 4.9 Prevention of adverse effects (Patient-specific Processes; Outcome) | No relevant sex differences in toxicity or survival were observed in this cohort; this is a null finding, not evidence of equivalence. | No sex difference in toxicity or survival was detected, so this cohort does not support sex-specific adaptation of palliative thoracic radiotherapy; equivalence was not established. |  |
| Frehner (2025) [21] | Pooled analysis of randomized trials; n = 499 | Sex | Multimodal; RT effect not separable. Pooled neoadjuvant regimens differed and did not isolate a radiotherapy effect. | 4.9 Prevention of adverse effects (Patient-specific Processes; Outcome) | Treatment-related grade 3 or higher adverse events and treatment discontinuation did not differ significantly between women and men. This is a null toxicity finding, not evidence of equivalence. | No sex difference in severe adverse events or treatment discontinuation was detected; this does not establish equivalence or justify different preventive care. | 4.13 |
| Zwart (2022) [22] | Retrospective cohort; n = 193 | Sex | Mixed treatment; RT subgroup. The cohort included surgery and (chemo)radiotherapy; adverse-event analyses relevant to RT used the (C)RT subgroup. | 4.9 Prevention of adverse effects (Patient-specific Processes; Outcome) | Sex-specific skeletal muscle index cut-offs were derived. In the 135-patient (chemo)radiotherapy subgroup, low muscle index was independently associated with grade ≥3 toxicity. This does not establish an independent female/male toxicity difference. | Low skeletal muscle index, which differed by sex, identified patients at increased risk of severe toxicity and may improve pretreatment risk assessment beyond sex alone. |  |
| Wolff (2011) [25] | Prospective cohort; n = 196 | Unclear | Multimodal; RT effect not separable. Toxicity occurred during intensified radiochemotherapy; chemotherapy and RT contributions cannot be separated. | 4.9 Prevention of adverse effects (Patient-specific Processes; Outcome) | Acute toxicity during intensified rectal radiochemotherapy differed by sex and BMI; all grade 4 toxicities occurred in women with low BMI, while hematologic toxicity did not differ significantly by sex. | Higher acute toxicity among women, particularly women with low body mass index, supports sex- and body-composition-aware monitoring during intensified rectal chemoradiotherapy. |  |
| Kaminski (2007) [47] | Prospective cohort; n = 84 | Unclear | Multimodal; RT effect not separable. Chemoradiotherapy was delivered, so the radiotherapy-specific contribution is not separable. | 4.9 Prevention of adverse effects (Patient-specific Processes; Outcome) | Acute toxicity was greater among women in this treatment-specific cohort. | Greater acute toxicity among women supports sex-stratified monitoring and supportive care during rectal chemoradiotherapy; a tailored preventive intervention was not evaluated. |  |
| Sekine (2009) [48] | Retrospective cohort; n = 204 | Unclear | Multimodal; RT effect not separable. Concurrent chemoradiotherapy was delivered, so the radiotherapy-specific contribution is not separable. | 4.9 Prevention of adverse effects (Patient-specific Processes; Outcome) | No female/male difference was observed for the reported severe toxicities. | No sex difference in the reported severe toxicities was observed, providing no signal for sex-specific monitoring in this cohort; equivalence was not established. | 4.13 |
| Dinapoli (2026) [23] | Retrospective cohort; n = 895 | Unclear | RT care process. Psychosocial findings were measured during radiation-oncology care; they are not radiation dose effects. | 4.12 Symptom management (Patient-specific Processes; Outcome) | Women reported greater distress, anxiety, depression, and voluntary psychosocial-support use overall, while no sex difference was found for rectal/anal cancers; results were diagnosis-dependent. | Diagnosis-dependent excess distress and greater psychosocial-support use among women support routine, sex-aware psychosocial assessment rather than assumptions based on sex alone. |  |
| Dinapoli (2026) [23] | Retrospective cohort; n = 895 | Unclear | RT care process. Psychosocial findings were measured during radiation-oncology care; they are not radiation dose effects. | 4.12 Symptom management (Patient-specific Processes; Outcome) | No significant association was observed between radiotherapy toxicity level and distress, anxiety, or depression. | The absence of an association between physical toxicity and psychological symptoms indicates that psychosocial distress requires direct assessment and cannot be inferred from toxicity severity. |  |
| Man and Ho (2025) [24] | Cross-sectional survey; n = 114 | Unclear | Post-RT/survivorship. Cross-sectional mental-health findings were assessed after completion of radiotherapy. | 4.12 Symptom management (Patient-specific Processes; Outcome) | In a cross-sectional post-radiotherapy sample, men showed poorer mental-health measures in an unadjusted model; associations with depression and stress were attenuated after optimism was included. No radiotherapy-causal or predictive effect was established. | Post-radiotherapy mental-health differences support sex-stratified psychosocial screening, but cross-sectional data do not establish a radiotherapy-related causal effect. |  |
| Sheehan (2019) [26] | Prospective registry cohort; n = 116 | Sex | RT-specific. Quality-of-life change was evaluated after stereotactic radiosurgery. | 4.12 Symptom management (Patient-specific Processes; Outcome) | "Male sex predicted a risk of worsening (average of 0.347) of the pain and discomfort subscore at last follow-up (p = 0.004205)... Sex differences appear to exist regarding pain and discomfort worsening after SRS." | Sex-associated worsening of pain and discomfort after stereotactic radiosurgery supports anticipatory counseling and symptom monitoring, without establishing a sex-specific treatment modification. |  |
| Bohm (2024) [27] | Prospective cohort; n = 75 | Sex | Mixed treatment/survivorship. Head-and-neck quality-of-life trajectories followed curative oncologic treatment; RT-specific attribution is limited. | 4.12 Symptom management (Patient-specific Processes; Outcome) | During treatment, men reported more pain-related burden and need for pain medication or nutritional support, whereas women reported more weight loss and dry mouth. Three months after treatment, women reported more problems, particularly dry mouth, weight loss, and sticky saliva. | Different symptom trajectories during and after head-and-neck treatment support sex-stratified surveillance of pain, nutrition, weight loss, xerostomia, and saliva-related symptoms. | 4.13 |
| Palazzi (2008) [28] | Prospective observational cohort; n = 149 | Unclear | RT-centered multimodal. Acute toxicity was observed during radiotherapy, often with concomitant chemotherapy. | 4.12 Symptom management (Patient-specific Processes; Outcome) | The following variables emerged as independent factors predicting for a worse outcome: for mucositis, concomitant CHT (p < 0.001), and previous surgery (p < 0.05); for weight loss, accelerated RT (p = 0.002), concomitant CHT (p = 0.004), and older age (p = 0.03); for dysphagia, concomitant CHT (p = 0.002); for pain, female gender (p = 0.02); | Female sex independently predicted worse pain, supporting proactive pain assessment and supportive care during head-and-neck radiotherapy. |  |
| Schuster (2022) [40] | Prospective cohort; n = 710 | Unclear | Multimodal; RT effect not separable. Patients received radiochemotherapy; radiation and systemic-treatment effects cannot be separated. | 4.12 Symptom management (Patient-specific Processes; Outcome) | Symptom and laboratory patterns were heterogeneous and time-dependent. | Heterogeneous, time-dependent symptom and laboratory patterns support repeated, sex-stratified assessment rather than a single uniform monitoring schedule. | 4.3 |
| Chow (2018) [43] | Secondary analysis of a randomized trial; n = 847 | Unclear | RT-specific. Palliative re-irradiation was randomized, but subgroup analyses were secondary. | 4.12 Symptom management (Patient-specific Processes; Outcome) | Analgesic response was similar, while selected patient-reported changes were mixed. | Similar analgesic response with mixed patient-reported changes suggests that pain response should be assessed directly and not inferred from sex alone. | 4.6 |
| Chow (2017) [44] | Secondary analysis of a randomized trial; n = 298 | Unclear | RT-specific. All patients received palliative radiotherapy; sex/gender analyses were secondary and not randomized. | 4.12 Symptom management (Patient-specific Processes; Outcome) | Analgesic response was similar, while selected psychosocial changes were mixed. | Similar analgesic response but mixed psychosocial changes support combined symptom and psychosocial follow-up rather than sex-based assumptions about benefit. | 4.6 |
| Lapid (2013) [46] | Secondary analysis of a randomized trial; n = 131 | Unclear | Mixed oncology treatment. The supportive-care intervention was not specific to radiotherapy and subgroup analyses were exploratory. | 4.12 Symptom management (Patient-specific Processes; Process) | Exploratory subgroup responses were time- and domain-dependent and did not establish a persistent sex/gender-specific intervention effect. | Time- and domain-dependent quality-of-life responses do not support a uniform sex-specific intervention, but justify sex-stratified monitoring in supportive-care studies. | 4.6 |
| Heller (2025) [14] | Retrospective single-center cohort; n = 94 | Unclear | RT-centered; attribution limited. Thoracic RT was central, but the heart-failure association was not shown to be dose-mediated and adjustment for cardiotoxic systemic therapy was limited. | 4.13 Therapy completion and follow-up care (Patient-specific Processes; Outcome) | Women had higher unadjusted odds of post-RT heart failure. The analysis did not establish atrial-dose mediation, and multivariable control of cardiotoxic systemic therapy was limited. | Higher post-radiotherapy heart-failure odds in women support sex-stratified cardiac risk assessment and follow-up; dose mediation and treatment-specific causality were not established. | 4.3 |
| Wang (2025) [17] | Retrospective two-center cohort; n = 1118 | Sex | Mixed-modality comparison. The study compares surgery and stereotactic radiosurgery; selection and outcome effects may interact. | 4.13 Therapy completion and follow-up care (Patient-specific Processes; Outcome) | Women had a higher observed recurrence rate after SRS; the study does not establish that a sex-specific surveillance schedule improves outcomes. | Higher observed recurrence after stereotactic radiosurgery supports examining sex in surveillance risk models, but does not establish that different follow-up schedules improve outcomes. | 4.6 |
| Agolli (2025) [18] | Retrospective cohort; n = 309 | Sex | RT-centered multimodal. Definitive/adjuvant radiotherapy was central, but systemic treatment and disease factors may contribute. | 4.13 Therapy completion and follow-up care (Patient-specific Processes; Outcome) | Central high-dose nodal failures were more frequent in women than men (64.3% vs 28.8%; p=0.014), representing a recurrence-pattern difference distinct from the dermatitis finding. | Sex-related differences in high-dose nodal recurrence patterns may inform risk-stratified follow-up and target-failure review; causality and intervention benefit were not tested. | 4.9 |
| Frehner (2025) [21] | Pooled analysis of randomized trials; n = 499 | Sex | Multimodal; RT effect not separable. Pooled neoadjuvant regimens differed and did not isolate a radiotherapy effect. | 4.13 Therapy completion and follow-up care (Patient-specific Processes; Outcome) | Women had longer event-free and overall survival and lower non-cancer mortality. The heterogeneous multimodal regimens do not permit attribution to radiotherapy. | Sex-associated survival differences in heterogeneous multimodal treatment may refine prognostic counseling but do not support radiotherapy-specific management changes. | 4.9 |
| Bohm (2024) [27] | Prospective cohort; n = 75 | Sex | Mixed treatment/survivorship. Head-and-neck quality-of-life trajectories followed curative oncologic treatment; RT-specific attribution is limited. | 4.13 Therapy completion and follow-up care (Patient-specific Processes; Outcome) | At three months after treatment, women reported a greater burden of several persistent head-and-neck symptoms, while men had shown a higher need for nutritional support during treatment. | Persistent xerostomia, nutritional, weight, pain, and saliva-related differences support sex-stratified follow-up after head-and-neck treatment. | 4.12 |
| Myrehaug (2008) [29] | Retrospective cohort; n = 615 | Sex | Multimodal; RT effect not separable. Late cardiac morbidity reflects mediastinal RT combined with doxorubicin exposure. | 4.13 Therapy completion and follow-up care (Patient-specific Processes; Outcome) | Older age, male sex and the presence of diabetes were also significant risk factors for adverse cardiac events (p < 0.05). | Male sex was associated with late cardiac events after Hodgkin lymphoma treatment, supporting sex-stratified long-term cardiac surveillance alongside established risk factors. |  |
| Hertzberg (1997) [30] | Retrospective multicenter study; n = 118 | Sex | Multimodal; RT effect not separable. Childhood ALL treatment combined cranial irradiation and methotrexate-based therapy. | 4.13 Therapy completion and follow-up care (Patient-specific Processes; Outcome) | CNS alterations were not sex-related; combined cranial irradiation and methotrexate-based treatment increased morphological brain alteration risk. | The absence of a sex association with central nervous system alterations does not support sex-based decisions about cranial irradiation in this cohort; combined treatment exposure remained the relevant risk signal. |  |
| Waber (1990) [31] | Retrospective cohort; n = 66 | Sex | Multimodal; RT effect not separable. Late effects followed CNS treatment combining cranial irradiation and intrathecal therapy. | 4.13 Therapy completion and follow-up care (Patient-specific Processes; Outcome) | "Sex was the pre-eminent risk factor for CNS toxicity, females being more severely affected. Specifically, cognitive impairment was more prevalent and severe in females in the ALL sample, as was short stature and excessive weight-gain." | Greater cognitive, growth, and weight-related late effects among girls support sex-sensitive long-term surveillance after cranial irradiation and consideration of sex in risk-benefit discussions. |  |
| Tonning Olsson (2014) [32] | Retrospective cohort; n = 69 | Sex | Mixed treatment context. Late cognition was associated with several tumor and treatment factors, including whole-brain RT. | 4.13 Therapy completion and follow-up care (Patient-specific Processes; Outcome) | "Lowered IQ was associated with young age at diagnosis, being male, tumor size, and treatment with whole-brain radiation therapy. [...] Tumor size was found to be a better predictor of cognitive sequelae than sex." | Cognitive-risk assessment after pediatric brain-tumor therapy should consider sex jointly with tumor size, age, and whole-brain irradiation rather than apply a simple female-risk assumption. |  |
| Mangesius (2024) [33] | Retrospective cohort; n = 108 | Sex | RT-specific. Outcomes were evaluated after stereotactic radiosurgery. | 4.13 Therapy completion and follow-up care (Patient-specific Processes; Outcome) | Female sex was associated with longer observed survival after SRS. This observational association does not establish a sex-specific treatment or follow-up intervention. | The survival association after stereotactic radiosurgery may inform prognostic stratification, but does not establish a sex-specific treatment or surveillance intervention. |  |
| Luo (2019) [34] | Retrospective propensity-score-matched cohort; n = 683 | Sex | RT-specific. The cohort received definitive radiotherapy for esophageal cancer. | 4.13 Therapy completion and follow-up care (Patient-specific Processes; Outcome) | In the matched cohort, female patients still had a significantly longer median PFS (13.5 months vs 11.6 months) and OS (19.6 months vs 16.1 months). Multivariate analysis showed that sex was an independent prognostic factor for PFS. […] after a median follow-up of 16.6 months (range, 1.6 to 112.5 months), the logoregional recurrence rate in female patients was significantly lower than that in men (χ2 = 11.11, P = 0.0009). A similar finding was observed for distant metastasis event, with fewer patients experiencing distant metastases in female group than in male group. | Inferior progression and survival outcomes among men support evaluating sex within prognostic and follow-up models; the observational study does not demonstrate benefit from more intensive surveillance. |  |
| Mauch (1996) [35] | Retrospective cohort; n = 794 | Sex | Historical multimodal treatment. Second malignancy risk followed historical Hodgkin treatment with large radiation fields and other therapies. | 4.13 Therapy completion and follow-up care (Patient-specific Processes; Outcome) | Women treated at a young age had higher risks of breast and other solid second malignancies, while men showed different secondary-malignancy patterns; historical treatment fields limit current generalizability. | Sex-specific second-malignancy patterns support tailored long-term screening after historical Hodgkin lymphoma radiotherapy, including breast surveillance after young-age thoracic exposure. |  |
| Bhatia (2002) [36] | Retrospective cohort; n = 47 | Sex | RT-specific. Cataract risk was evaluated after curative orbital radiotherapy. | 4.13 Therapy completion and follow-up care (Patient-specific Processes; Outcome) | Men were more likely to develop cataracts than were women (p = 0.023). None of the following parameters was significantly associated with cataract formation: RT dose, fraction size, beam energy, beam arrangement, use of steroids, and use of lens block | Higher cataract incidence among men supports long-term ocular surveillance, although the study did not identify a dose or planning factor that explained the difference. |  |
| Bjordal (2001) [37] | Prospective cohort; n = 249 | Sex | Mixed treatment/survivorship. Longitudinal head-and-neck outcomes followed multimodal treatment; RT-specific attribution is limited. | 4.13 Therapy completion and follow-up care (Patient-specific Processes; Outcome) | Women and men showed different longitudinal head-and-neck symptom and quality-of-life trajectories after treatment. | Different longitudinal symptom and quality-of-life trajectories support sex-stratified follow-up of xerostomia, nutrition, and functional recovery after head-and-neck treatment. |  |
| Besserer (2026) [38] | Retrospective cohort; n = 2727 | Unclear | Post-RT/survivorship. The outcome is rehabilitation use after radiotherapy. | 4.13 Therapy completion and follow-up care (Patient-specific Processes; Process) | "Females accessed rehabilitation 2.6 times more frequently than males (25.1% vs. 9.6%, p<0.001) [...] Even with standardized information and administrative support, significant gender-specific and sociodemographic differences persist." | Lower rehabilitation use among eligible men despite standardized access indicates a sex/gender-related follow-up care gap and supports targeted referral and uptake monitoring. |  |
| Martling (2009) [45] | Retrospective cohort; n = 11774 | Unclear | Historical treatment selection. Treatment receipt and outcomes reflect historical registry practice and residual confounding. | 4.13 Therapy completion and follow-up care (Patient-specific Processes; Outcome) | Local-control benefit was present in both groups despite a treatment-receipt difference. | Benefit in local control despite different treatment receipt cautions against interpreting receipt differences alone as inequitable care and supports indication-adjusted audit. | 4.6 |
| Sekine (2009) [48] | Retrospective cohort; n = 204 | Unclear | Multimodal; RT effect not separable. Concurrent chemoradiotherapy was delivered, so the radiotherapy-specific contribution is not separable. | 4.13 Therapy completion and follow-up care (Patient-specific Processes; Outcome) | A response-rate difference did not translate into a survival difference. | A response-rate difference without a survival difference cautions against using early response alone to justify sex-specific follow-up or treatment changes. | 4.9 |
| Baumann (2006) [49] | Retrospective chart review and QoL survey; n = 34 | Unclear | Mixed treatment/survivorship. Quality of life followed surgery and postoperative radiotherapy; attribution to radiotherapy alone is not possible. | 4.13 Therapy completion and follow-up care (Patient-specific Processes; Outcome) | Follow-up quality-of-life differences were observed, with important background-population confounding. | Follow-up quality-of-life differences warrant sex-stratified supportive assessment, but background-population differences limit attribution to treatment. |  |
| Tariq (2014) [50] | Retrospective cohort; n = 1116 | Unclear | RT-centered; attribution limited. The association was univariable and thyroid dose, baseline function, age, and co-treatment were not jointly modeled. | 4.13 Therapy completion and follow-up care (Patient-specific Processes; Outcome) | Female group membership was associated with hypothyroidism in an univariable analysis. | The univariable association with hypothyroidism is a screening signal for sex-stratified thyroid follow-up, not evidence for a sex-specific monitoring protocol. |  |
| Käsmann (2025) [39] | Prospective cohort; n = 112 | Sex | RT care process. CAM use and interaction risk were assessed in patients undergoing radiotherapy. | 4.14 Monitoring and adjustment of non-tumor-targeting medication therapies (Patient-specific Processes; Outcome) | Women more often reported CAM use with possible or known interaction potential. The study did not demonstrate that an interaction or adverse drug event actually occurred. | More frequent use of potentially interacting complementary and alternative medicine among women supports systematic medication and CAM history-taking during radiotherapy; actual adverse interactions were not demonstrated. |  |

**Supplementary Table S2B. Complete patient-safety framework coverage**

*All 27 published subcategories are shown. Counts describe the distribution of retrieved evidence, not evidence strength or clinical importance.*

| **Code** | **Domain** | **Subcategory** | **Working operational boundary** | **Findings, n** | **Studies, n** | **Status** |
| --- | --- | --- | --- | --- | --- | --- |
| 1.1 | Human Resources | Promotion of continuous education and competency review | Apply the published framework definition; no included evidence is currently mapped here. | 0 | 0 | Unpopulated |
| 1.2 | Human Resources | Staffing | Apply the published framework definition; no included evidence is currently mapped here. | 0 | 0 | Unpopulated |
| 1.3 | Human Resources | Training: Courses for new technologies and procedures | Apply the published framework definition; no included evidence is currently mapped here. | 0 | 0 | Unpopulated |
| 2.1 | Institutional Culture | Promotion of a safety culture | Apply the published framework definition; no included evidence is currently mapped here. | 0 | 0 | Unpopulated |
| 2.2 | Institutional Culture | Internal feedback loops | Apply the published framework definition; no included evidence is currently mapped here. | 0 | 0 | Unpopulated |
| 2.3 | Institutional Culture | Patient involvement | Apply the published framework definition; no included evidence is currently mapped here. | 0 | 0 | Unpopulated |
| 3.1 | Quality and Risk Management | Existence and effectiveness of a system for error reporting and analysis | Apply the published framework definition; no included evidence is currently mapped here. | 0 | 0 | Unpopulated |
| 3.2 | Quality and Risk Management | External peer review procedures | Apply the published framework definition; no included evidence is currently mapped here. | 0 | 0 | Unpopulated |
| 3.3 | Quality and Risk Management | Internal peer review procedures | Apply the published framework definition; no included evidence is currently mapped here. | 0 | 0 | Unpopulated |
| 3.4 | Quality and Risk Management | Control points and checklists | Apply the published framework definition; no included evidence is currently mapped here. | 0 | 0 | Unpopulated |
| 3.5 | Quality and Risk Management | Failure of therapy-critical processes | Apply the published framework definition; no included evidence is currently mapped here. | 0 | 0 | Unpopulated |
| 3.6 | Quality and Risk Management | Standard Operating Procedures (SOPs) | Apply the published framework definition; no included evidence is currently mapped here. | 0 | 0 | Unpopulated |
| 3.7 | Quality and Risk Management | Technology assessment and risk analyses for new procedures | Apply the published framework definition; no included evidence is currently mapped here. | 0 | 0 | Unpopulated |
| 4.1 | Patient-specific Processes | Selection and administration of tumor-effective systemic therapies | Apply the published framework definition; no included evidence is currently mapped here. | 0 | 0 | Unpopulated |
| 4.2 | Patient-specific Processes | Treatment of patients with pacemakers and defibrillators | Apply the published framework definition; no included evidence is currently mapped here. | 0 | 0 | Unpopulated |
| 4.3 | Patient-specific Processes | Radiation planning and plan evaluation | Sex/gender differences directly involving dosimetry, constraints, prescription, plan optimization, or plan evaluation. Toxicity alone does not demonstrate a planning difference. | 3 | 3 | Populated |
| 4.4 | Patient-specific Processes | Contouring | Sex/gender differences in target/OAR delineation, contouring rules, or contour quality. Dose constraints belong to 4.3. | 0 | 0 | Unpopulated |
| 4.5 | Patient-specific Processes | Initial setup and subsequent irradiations | Apply the published framework definition; no included evidence is currently mapped here. | 0 | 0 | Unpopulated |
| 4.6 | Patient-specific Processes | Patient presentation, informed consent, indication and prescription | Differences in presentation, access, modality choice, indication, risk communication, consent, prescription, or directly relevant prognostic assessment. | 8 | 8 | Populated |
| 4.7 | Patient-specific Processes | Patient-specific quality assurance | Apply the published framework definition; no included evidence is currently mapped here. | 0 | 0 | Unpopulated |
| 4.8 | Patient-specific Processes | Planning imaging and positioning | Apply the published framework definition; no included evidence is currently mapped here. | 0 | 0 | Unpopulated |
| 4.9 | Patient-specific Processes | Prevention of adverse effects | Preventive action before an adverse effect occurs. Observed toxicity is coded here only as outcome evidence with an explicit prevention implication, not as proof of preventive care. | 8 | 8 | Populated |
| 4.10 | Patient-specific Processes | Spatial and temporal organization of patient pathways | Apply the published framework definition; no included evidence is currently mapped here. | 0 | 0 | Unpopulated |
| 4.11 | Patient-specific Processes | Interfaces between care providers | Apply the published framework definition; no included evidence is currently mapped here. | 0 | 0 | Unpopulated |
| 4.12 | Patient-specific Processes | Symptom management | Recognition, monitoring, or treatment of physical or psychosocial symptoms during or near radiotherapy. Long-term surveillance belongs to 4.13. | 10 | 9 | Populated |
| 4.13 | Patient-specific Processes | Therapy completion and follow-up care | Treatment completion, rehabilitation, follow-up, survivorship, or screening for late effects. Survival or recurrence alone does not prove a follow-up difference. | 19 | 19 | Populated |
| 4.14 | Patient-specific Processes | Monitoring and adjustment of non-tumor-targeting medication therapies | Medication history, CAM, supportive/non-oncologic medication, interactions, monitoring, or adjustment. Tumor-effective systemic therapy belongs to 4.1. | 1 | 1 | Populated |
