# Supplementary material for: Sex- and gender-related aspects of patient safety in radiation oncology: a scoping review
Source: Front Oncol. 2026 Oct 27;16:1962214. doi: 10.3389/fonc.2026.1962214 (PMC13749819; doi:10.3389/fonc.2026.1962214)
Supplement: Supplementary file 2 [file Table2.docx]

**Preferred Reporting Items for Systematic reviews and Meta-Analyses extension for Scoping Reviews (PRISMA-ScR) Checklist**

Manuscript: Sex- and gender-related aspects of patient safety in radiation oncology: a scoping review

Updated: 1 October 2026

| **SECTION** | **ITEM** | **PRISMA-ScR CHECKLIST ITEM** | **REPORTED IN SECTION / TABLE** |
| --- | --- | --- | --- |
| **TITLE** | | | |
| Title | 1 | Identify the report as a scoping review. | (title). |
| **ABSTRACT** | | | |
| Structured summary | 2 | Provide a structured summary that includes (as applicable): background, objectives, eligibility criteria, sources of evidence, charting methods, results, and conclusions that relate to the review questions and objectives. | (structured Abstract; final search and 37-study synthesis). |
| **INTRODUCTION** | | | |
| Rationale | 3 | Describe the rationale for the review in the context of what is already known. Explain why the review questions/objectives lend themselves to a scoping review approach. | (Introduction). |
| Objectives | 4 | Provide an explicit statement of the questions and objectives being addressed with reference to their key elements (e.g., population or participants, concepts, and context) or other relevant key elements used to conceptualize the review questions and/or objectives. | (final paragraph of Introduction). |
| **METHODS** | | | |
| Protocol and registration | 5 | Indicate whether a review protocol exists; state if and where it can be accessed (e.g., a Web address); and if available, provide registration information, including the registration number. | (Methods 2.1; no review protocol was registered or published). |
| Eligibility criteria | 6 | Specify characteristics of the sources of evidence used as eligibility criteria (e.g., years considered, language, and publication status), and provide a rationale. | (Methods 2.4 and Table 1). |
| Information sources* | 7 | Describe all information sources in the search (e.g., databases with dates of coverage and contact with authors to identify additional sources), as well as the date the most recent search was executed. | (Methods 2.2-2.3; PubMed searched on 22 July 2026 and backward citation searching). |
| Search | 8 | Present the full electronic search strategy for at least 1 database, including any limits used, such that it could be repeated. | (Methods 2.2); Supplementary Table S1 (complete normalized PubMed query and search metadata). |
| Selection of sources of evidence† | 9 | State the process for selecting sources of evidence (i.e., screening and eligibility) included in the scoping review. | (Methods 2.5; four-reviewer calibration of 15 records within the 159-record PubMed set and remaining single-reviewer workflow). |
| Data charting process‡ | 10 | Describe the methods of charting data from the included sources of evidence (e.g., calibrated forms or forms that have been tested by the team before their use, and whether data charting was done independently or in duplicate) and any processes for obtaining and confirming data from investigators. | (Methods 2.6-2.7; finding-level charting, classification, framework mapping, team resolution of uncertainty, and no contact with primary-study investigators). |
| Data items | 11 | List and define all variables for which data were sought and any assumptions and simplifications made. | (Methods 2.6-2.7); Supplementary Table S2 (full study-finding-category matrix). |
| Critical appraisal of individual sources of evidence§ | 12 | If done, provide a rationale for conducting a critical appraisal of included sources of evidence; describe the methods used and how this information was used in any data synthesis (if appropriate). | Not applicable: no formal critical appraisal was performed (Methods 2.8). |
| Synthesis of results | 13 | Describe the methods of handling and summarizing the data that were charted. | (Methods 2.7-2.8). |
| **RESULTS** | | | |
| Selection of sources of evidence | 14 | Give numbers of sources of evidence screened, assessed for eligibility, and included in the review, with reasons for exclusions at each stage, ideally using a flow diagram. | (Results 3.1 and Figure 2; 159 records screened, 41 PubMed reports assessed, and 37 primary studies included). |
| Characteristics of sources of evidence | 15 | For each source of evidence, present characteristics for which data were charted and provide the citations. | (Results 3.2 and Table 2; characteristics of all 37 included studies). |
| Critical appraisal within sources of evidence | 16 | If done, present data on critical appraisal of included sources of evidence (see item 12). | Not applicable: no formal critical appraisal was performed (Methods 2.8). |
| Results of individual sources of evidence | 17 | For each included source of evidence, present the relevant data that were charted that relate to the review questions and objectives. | (Results 3.3-3.8); Table 2; Supplementary Table S2A (49 findings from 37 studies). |
| Synthesis of results | 18 | Summarize and/or present the charting results as they relate to the review questions and objectives. | (Results 3.3-3.8 and Figure 1 and Supplementary Material S2); Supplementary Table S2B. |
| **DISCUSSION** | | | |
| Summary of evidence | 19 | Summarize the main results (including an overview of concepts, themes, and types of evidence available), link to the review questions and objectives, and consider the relevance to key groups. | (Discussion 4.1-4.6). |
| Limitations | 20 | Discuss the limitations of the scoping review process. | (Discussion 4.6, Limitations). |
| Conclusions | 21 | Provide a general interpretation of the results with respect to the review questions and objectives, as well as potential implications and/or next steps. | (Conclusions). |
| **FUNDING** | | | |
| Funding | 22 | Describe sources of funding for the included sources of evidence, as well as sources of funding for the scoping review. Describe the role of the funders of the scoping review. | (funding of included studies was not systematically charted; the scoping review received no specific funding and therefore had no funder role). |

JBI = Joanna Briggs Institute; PRISMA-ScR = Preferred Reporting Items for Systematic reviews and Meta-Analyses extension for Scoping Reviews.

* Where *sources of evidence* (see second footnote) are compiled from, such as bibliographic databases, social media platforms, and Web sites.

† A more inclusive/heterogeneous term used to account for the different types of evidence or data sources (e.g., quantitative and/or qualitative research, expert opinion, and policy documents) that may be eligible in a scoping review as opposed to only studies. This is not to be confused with *information sources* (see first footnote).

‡ The frameworks by Arksey and O’Malley (6) and Levac and colleagues (7) and the JBI guidance (4, 5) refer to the process of data extraction in a scoping review as data charting*.*

§ The process of systematically examining research evidence to assess its validity, results, and relevance before using it to inform a decision. This term is used for items 12 and 19 instead of "risk of bias" (which is more applicable to systematic reviews of interventions) to include and acknowledge the various sources of evidence that may be used in a scoping review (e.g., quantitative and/or qualitative research, expert opinion, and policy document).

*From:* Tricco AC, Lillie E, Zarin W, O'Brien KK, Colquhoun H, Levac D, et al. PRISMA Extension for Scoping Reviews (PRISMAScR): Checklist and Explanation. Ann Intern Med. 2018;169:467–473. [doi: 10.7326/M18-0850](http://annals.org/aim/fullarticle/2700389/prisma-extension-scoping-reviews-prisma-scr-checklist-explanation).
